# Membrane guanylate cyclase, a multimodal transduction machine: history, present, and future directions

**DOI:** 10.3389/fnmol.2014.00056

**Published:** 2014-07-02

**Authors:** Rameshwar K. Sharma, Teresa Duda

**Affiliations:** Research Divisions of Biochemistry and Molecular Biology, The Unit of Regulatory and Molecular Biology, Salus UniversityElkins Park, PA, USA

**Keywords:** membrane guanylate cyclase, cyclic GMP, multiple transduction modes

## Abstract

A sequel to these authors' earlier comprehensive reviews which covered the field of mammalian membrane guanylate cyclase (MGC) from its origin to the year 2010, this article contains 13 sections. The first is historical and covers MGC from the year 1963–1987, summarizing its colorful developmental stages from its passionate pursuit to its consolidation. The second deals with the establishment of its biochemical identity. MGC becomes the transducer of a hormonal signal and founder of the peptide hormone receptor family, and creates the notion that hormone signal transduction is its sole physiological function. The third defines its expansion. The discovery of ROS-GC subfamily is made and it links ROS-GC with the physiology of phototransduction. Sections ROS-GC, a Ca^2+^-Modulated Two Component Transduction System to Migration Patterns and Translations of the GCAP Signals Into Production of Cyclic GMP are Different cover its biochemistry and physiology. The noteworthy events are that augmented by GCAPs, ROS-GC proves to be a transducer of the free Ca^2+^ signals generated within neurons; ROS-GC becomes a two-component transduction system and establishes itself as a source of cyclic GMP, the second messenger of phototransduction. Section ROS-GC1 Gene Linked Retinal Dystrophies demonstrates how this knowledge begins to be translated into the diagnosis and providing the molecular definition of retinal dystrophies. Section Controlled By Low and High Levels of [Ca^2+^]_i_, ROS-GC1 is a Bimodal Transduction Switch discusses a striking property of ROS-GC where it becomes a “[Ca^2+^]_i_ bimodal switch” and transcends its signaling role in other neural processes. In this course, discovery of the first CD-GCAP (Ca^2+^-dependent guanylate cyclase activator), the S100B protein, is made. It extends the role of the ROS-GC transduction system beyond the phototransduction to the signaling processes in the synapse region between photoreceptor and cone ON-bipolar cells; in section Ca^2+^-Modulated Neurocalcin δ ROS-GC1 Transduction System Exists in the Inner Plexiform Layer (IPL) of the Retinal Neurons, discovery of another CD-GCAP, NCδ, is made and its linkage with signaling of the inner plexiform layer neurons is established. Section ROS-GC Linkage With Other Than Vision-Linked Neurons discusses linkage of the ROS-GC transduction system with other sensory transduction processes: Pineal gland, Olfaction and Gustation. In the next, section Evolution of a General Ca^2+^-Interlocked ROS-GC Signal Transduction Concept in Sensory and Sensory-Linked Neurons, a theoretical concept is proposed where “Ca^2+^-interlocked ROS-GC signal transduction” machinery becomes a common signaling component of the sensory and sensory-linked neurons. Closure to the review is brought by the conclusion and future directions.

## Introduction

**Figure 1:**
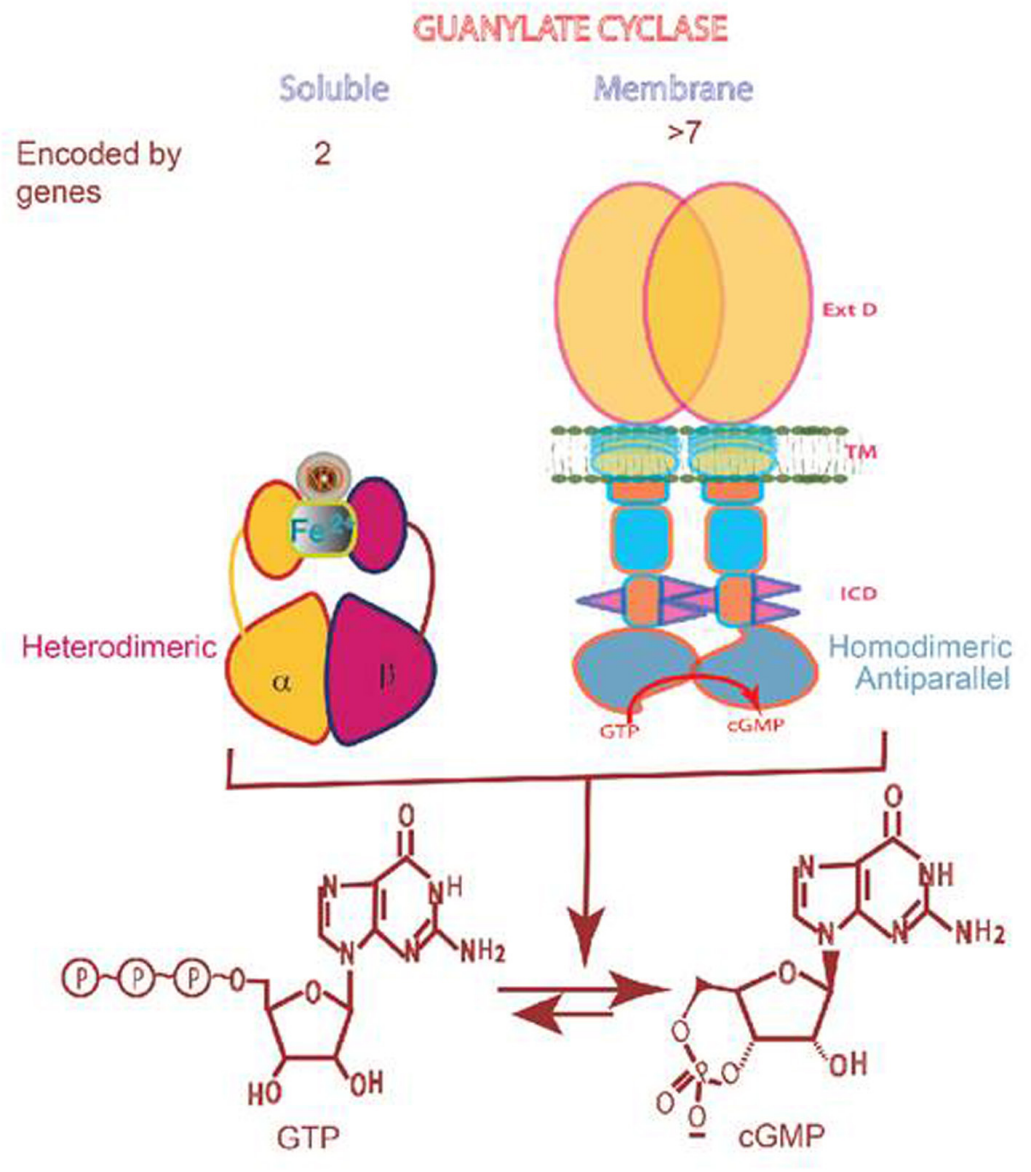
**Soluble vs membrane guanylate cyclase. Upper panel:** Graphical representation of soluble and membrane guanylate cyclases. The soluble form is encoded by two genes; it is heterodimeric (subunits α and β) and requires heme for its activity. Both monomers contribute to the catalytic center, but their orientation is unknown yet (Allerston et al., 2013). The membrane guanylate cyclase is encoded by 7 genes. It is a single transmembrane spanning protein. The extracelular domain (Ext) is located outside the cell; the transmembrane domain (TM) spans the plasma membrane; and the intracellular domain (ICD) is located inside the cell. The active form is homodimeric. The cyclase catalytic domain is located at the C-terminus of the protein. Both monomers in antiparallel orientation contribute to the catalytic center (Duda et al., 2012). **Lower panel:** Both cyclases are lyases (EC4.6.1.2) and catalyze synthesis of cyclic GMP from GTP.

In addition to cyclic AMP and inositol triphosphate (IP_3_), cyclic GMP is an omnipresent intracellular second messenger of prokaryotes and eukaryotes. It plays a critical role in the control of physiological processes of cardiac vasculature, smooth muscle relaxation, blood pressure, blood volume, cellular growth, sensory transduction, neural plasticity, learning, and memory. Unique to itself, this second messenger system is generated by the catalysis of GTP by two structurally different guanylate cyclases, soluble and the membrane form (Figure 1). The synthetic machinery and the modes of their operation by which they generate cyclic GMP are entirely different, and so are their physiologically regulated processes. This review is focused on the mammalian membrane guanylate cyclase transduction field, a principal area of research by the authors for several decades. It begins with its historical development. Briefly narrates the efforts involved building on small projects, brick by brick, and emerged from the chasm of disbelief, through steady, continuous work. It then moves on to its present status. To make the presentation simple and chronologically continuous, the subject matters of the earlier reviews (Sharma et al., 1997; Pugh et al., 1997; Sharma, 2002; Duda et al., 2005b; Sharma, 2010; Sharma and Duda, 2012) and publication of these authors have been freely borrowed with appropriate citations.

## History: year 1963–1987

### Background

The present day usage of the term “signal transduction” is rooted in the original discovery of cyclic AMP (Sutherland and Rall, 1958; reviewed in: Sutherland and Rall, 1960) for which Earl Wilbur Sutherland, Jr. won a Nobel Prize in physiology/medicine in 1971. Based on the studies with glycogen metabolism in skeletal muscle, Sutherland's group proposed the “second messenger” role of cyclic AMP in the hormone actions of epinephrine and glucagon (reviewed in Sharma et al., 1997). This “second messenger” concept was revolutionary because the consensus at the time was that the hormones acted directly on their target tissues, and, thus the hormonal pathway could only be studied in intact cells. Sutherland's studies demonstrated that it was possible to study the cell-free systems and monitor the generation of cyclic AMP indicative of the parent hormone action. Conceptually, the hormone action occurred in the particulate fraction of the cell in two stages; first, the hormone interacted with the particulate fraction of a cell and second, generated its second messenger, cyclic AMP.

In this concept, the hormone was the first messenger whose interaction with its receptor resulted in a stimulus that caused the production of the second messenger “cyclic AMP,” which then acted as the hormone messenger in eliciting the intracellular response (reviewed in Sharma et al., 1997). Thus, the term “second messenger” was coined. Reflective of the time, the biochemical nature of the receptor was not known, because none had been purified. It was not clear whether the enzyme, termed adenylate cyclase, catalyzing the formation of cyclic AMP, was a separate molecule or part, as a subunit, of the receptor. However, it was envisioned that interaction between the receptor and adenylate cyclase generates the second messenger. So enticing was the concept that it was viewed to be applicable to all hormonal systems, i.e., that cyclic AMP is the universal second messenger of all hormones (Robison et al., 1967). Hence, the general concept evolved in which hormone, the first messenger, transmits the extracellular (biological) information to the interior of the cellular machinery through the adenylate cyclase system, and the concept supposed that the receptor was a part of the adenylate cyclase system. The receptor was conceptualized, as defined by the studies in 1930s, as a part of the cell where hormone, the stimulus, interacts (Clark, 1937; Ariëns and Simonis, 1966). The term “stimulus” was equivalent to the presently used term “signal,” which, then, according to the “second messenger” concept meant that adenylate cyclase was the means to convert the extracellular ligand binding signal, into the production of the intracellular messenger cyclic AMP. Implicit in this concept was the understanding that the events leading to the transformation of the hormonal binding signal into the production of the second messenger occurred in the cell's plasma membrane.

**Figure 2:**
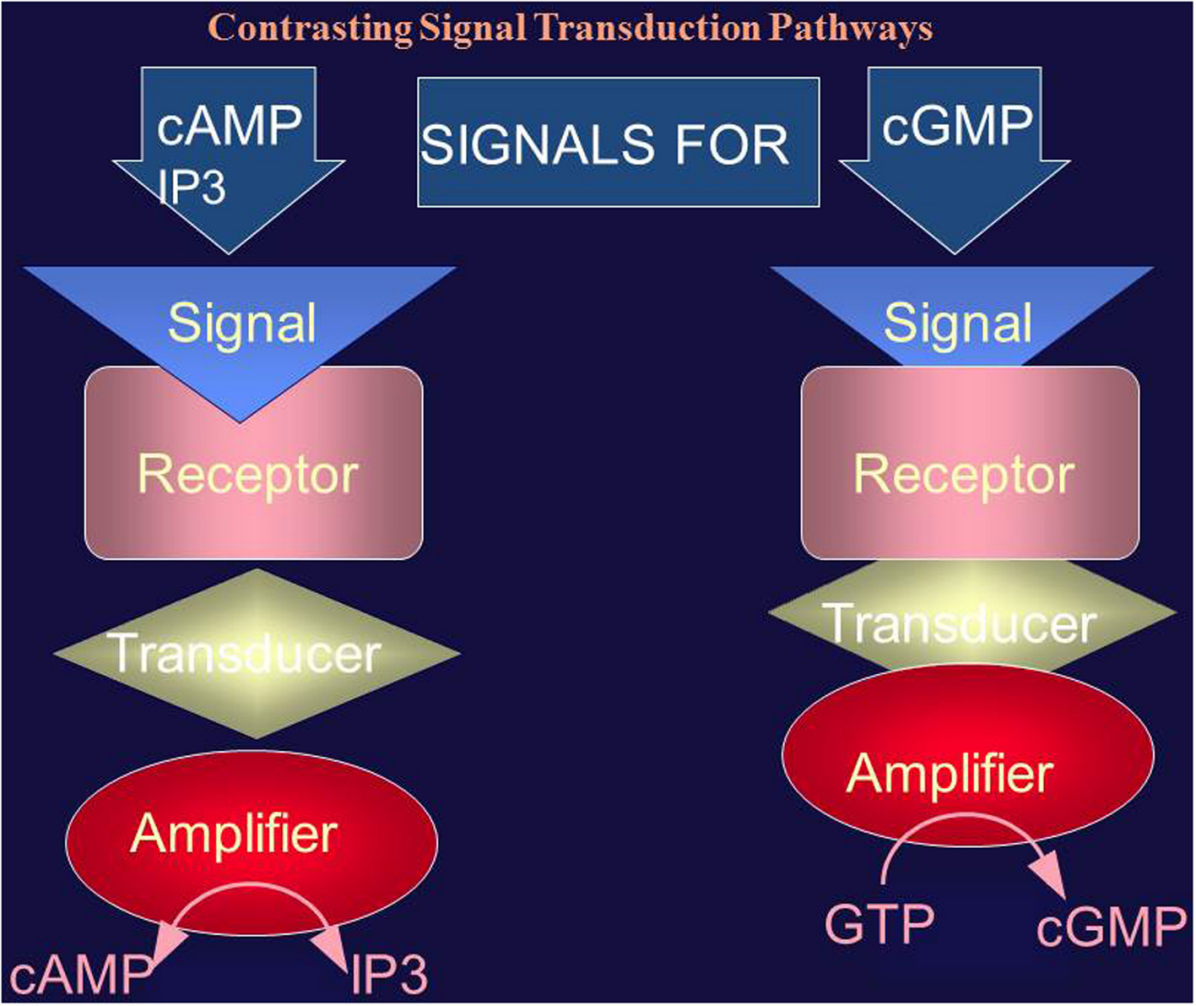
**Comparison of the cyclic AMP (and IP3) and cyclic GMP signaling systems**. The cyclic AMP (and IP3) signaling system consists of three separate components: hormone receptor, transducer (G protein) and amplifier (adenylate cyclase). The cyclic GMP signaling system consists of a single protein. The hormonal signal is recognized by the extracellular receptor domain; the signal is potentiated at the ATP-modulated ARM domain located next to the transmembrane domain in the intracellular portion of the protein (transducer) and the signal is amplified by the cyclase catalytic domain located at the C-terminus of the protein.

The “cyclic AMP second messenger concept” was brought to the molecular level by the discoveries of Martin Rodbell and Alfred G. Gilman for which in 1994 they shared the Nobel Prize in physiology/medicine. They demonstrated that G-protein signaling bridged the gap between the hormonal signal and activation of the adenylate cyclase. Rodbell's group argued that the cellular system that processes the hormonal signal was made up of three separate molecular entities: (1) Discriminator (RECEPTOR); (2) Transducer (G-Protein); (3) Amplifier, which as we understand now is the adenylate cyclase. The surface receptor receives information from the exterior of the cell; the G-protein (transducer) transmigrates the information across the cell membrane; and the amplifier (adenylate cyclase) strengthens it to initiate reactions within the cell or to communicate with other cells. In formulating this “G-Protein hormonal hypothesis” the group demonstrated that GTP was obligatory for the hormonal action of glucagon in liver membranes. The term TRANSDUCTION was introduced to indicate this GTP-dependent transformation step. And usage of the term SIGNAL TRANSDUCTION entered the field of cellular signaling (these early studies are reviewed in Rodbell, 1978). The Gilman's and then Birnbaumer's group defined the sequence of these signal transduction steps (Hepler and Gilman, 1992; Birnbaumer and Birnbaumer, 1995). Two types of G proteins, one stimulatory, termed G_s_, and the other inhibitory, G_i_, were purified and characterized; G_s_ mediated the stimulatory and G_i_ the inhibitory transduction. The nature of the receptor defined the specificity of the hormone. The cyclic AMP signaling system therefore comprised of three distinct proteins—receptor, G-protein, and adenylate cyclase (Figure 2).

Following the three-component signaling template of cyclic AMP, phosphatidylinositol (IP_3_) cellular signaling pathway evolved. Here, the surface receptor responded to an extracellular hormonal signal and converted phosphatidyl-4,5-bisphosphate (PIP_2_) in two separate second messengers, diacylglycerol and inositol trisphosphate (IP_3_). IP_3_ signal transduction splits into two: (1) mobilizes the intracellular calcium and (2) results in the activation of protein kinase C (Early studies respectively reviewed in Nishizuka, 1988; Berridge and Irvine, 1989).

In this manner, these, and complimentary studies from other laboratories (reviewed in Strader et al., 1994) established the presence of two major G-proteins linked with three-component cellular signal transduction pathways—cyclic AMP and phosphatidylinositol—providing the means by which the extracellular hormonal signal is translated into the production of the intracellular second messengers. These second messengers, in turn, initiate a cascade of chemical reactions which ultimately translate the hormonal signal into a biological response.

### Cyclic GMP signaling pathway

#### Pursuit

Besides cyclic AMP, could another cyclic nucleotide be an alternative hormonal second messenger in cellular signaling?

Cyclic GMP was first identified in rat urine (Ashman et al., 1963). Six years later, cyclic GMP and the enzyme membrane guanylate cyclase catalyzing its synthesis from GTP were detected in all tested animal tissues (Goldberg et al., 1969, 1973; Ishikawa et al., 1969). A groundbreaking “Ying Yang” hypothesis was proposed where both cyclic AMP and cyclic GMP served as hormonal second messengers. They were intertwined and exhibited opposing biological activities of the cellular regulation (Goldberg et al., 1975).

#### Denial

The celebration was short lived, however. The original proponents failed to consistently demonstrate a hormonally dependent guanylate cyclase in their tested systems (Goldberg and Haddox, 1977). They observed that a variety of non-hormonal ligands like polyunsaturated fatty acids, peroxides, hydroperoxides, free radicals, ascorbic acid, sodium nitroprusside, cigarette smoke also stimulated membrane guanylate cyclase activity. Thus, the membrane guanylate cyclase was a non-specific enzyme and underwent regulation *via* the oxidation-reduction potential of the biochemical reactions (Murad et al., 1979). Also, at the time, the only known cyclic GMP-dependent cellular component was cyclic GMP-dependent protein kinase but not one of its specific, distinct from that of the cyclic AMP-dependent protein kinase, substrate was known. Hence, the revised consensus was that the cyclic GMP-dependent system lacked specificity and acted as a sub-servant to the cyclic AMP system. Therefore, no distinct hormonally dependent membrane guanylate cyclase or cyclic GMP-related specific signaling system existed in any of the biological systems and cyclic GMP had no second messenger role in cellular signaling (Gill and McCune, 1979).

With a new twist, this view was strengthened as the presence of a soluble form of guanylate cyclase activity was found in the originally tested systems (Murad et al., 1979). This form was stimulated by polyunsaturated fatty acids, peroxides, hydroperoxides, free radicals and other agents. Impacted by these new findings and a passion to pursue this new field of soluble guanylate cyclase, the role of cyclic GMP in hormonal signal transduction was totally discarded (Goldberg and Haddox, 1977; Murad et al., 1979). These views are summed up in two of many high-impact reviews:

“While the metabolism of cyclic GMP has many parallelism to that of cyclic AMP, it is becoming quite clear that the two systems have distinct differences and that the functions of one system may not necessarily provide clues to the function of the other. In retrospect, the apparent parallelisms of the two systems hindered the originality of approaches to the cyclic GMP system and may, in fact, have impeded some of the developments in the area. Certainly, many early experiments with guanylate cyclase and cyclic GMP conducted in this laboratory and others were done based on previous experience and biases with the cyclic AMP system …, the properties of the soluble and particulate activities in tissues are quite different …. Minor differences have also been noted between particulate activities and in plasma and endoplasmic reticulum preparations. In spite of these significant physical and/or kinetic differences that suggest two or more forms of guanylate cyclase may exist in tissues, it is not yet known whether or not the proteins or their subunits are different. The apparent differences may be attributable to assay conditions, other constituents in crude preparations, or possibly other factors” (Murad et al., 1979).

“Considerable effort has been put forth to demonstrate an activation of the enzyme by hormonal or other physiological agents that promote the accumulation of cGMP in intact cells. To date, no specific direct effect of such an agent on guanylate cyclase activity *in vitro* has been convincingly demonstrated. This is a characteristic of the cGMP system that contrasts sharply with its cAMP counterpart; adenylate cyclase from disrupted cells as a general rule can be activated by the agents that stimulate cellular cAMP accumulation …. It is becoming evident that the greatest dissimilarity between guanylate cyclase and adenylate cyclase may be that the activity of the former is modulated indirectly with respect to the cellular action of hormones (and other cell stimuli) compared with the direct effect that hormones seem to have on the activity of adenylate cyclase” (Goldberg and Haddox, 1977).

#### Consolidation, parallel competing concept

The above intimidating concept, lasting to the mid-1980s, was challenged mainly by the competing concept of our group. It favored the hormonal second messenger role of cyclic GMP (reviewed in Sharma, 2010). Studies with the model systems of the isolated adrenal fasciculata and the adrenocortical carcinoma cells established a membrane guanylate cyclase to be the direct and specific transducer of the adrenocorticotropic hormone (ACTH). Notably, ACTH had no effect on the activity of the soluble guanylate cyclase (reviewed in Sharma, 2002). Thus, the guanylate cyclase existed in two forms, membrane and soluble; and the membrane form was hormone-dependent. These studies are briefly outlined below.

The models of the isolated adrenal fasciculata and the adrenocortical carcinoma cells were tailored to have no phosphodiesterase activity (Kitabchi and Sharma, 1971), and they were used to investigate the second messenger roles of cyclic AMP and cyclic GMP in steroidogenesis. The tailoring created a heightened membrane response to ACTH and it was direct, reflective of membrane-specific guanylate cyclase activity. The tailored PDE-deleted isolated fasciculata cells were sensitive to as little as 0.5–10 μU ACTH in stimulating corticosterone synthesis (Sharma et al., 1974, 1976; Perchellet et al., 1978). These concentrations of ACTH did not raise the level of cyclic AMP, yet they stimulated steroidogenesis (Sharma et al., 1974, 1976; Perchellet et al., 1978). The rise in cyclic AMP level was observed only at ACTH concentrations beyond their maximal steroidogenic capacity (Sharma et al., 1974). Thus, cyclic AMP was not the physiological second messenger of ACTH.

The physiological levels of ACTH in these cells also generated an excellent temporal correlation between the sequential production of cyclic GMP, phosphorylation, and the concomitant synthesis of corticosterone, indicating that the hormone caused the production of cyclic GMP, this, in turn, led to phosphorylation, and finally to corticosterone synthesis (Sharma et al., 1976, 1977). On similar lines, addition of cyclic GMP to these cells stimulated the protein kinase activity which was followed by a rise in their steroidogenic activity (Sharma et al., 1976); and, importantly, this added cyclic GMP mimicked the exogenously supplied ACTH action in stimulating the direct transformation of cholesterol to corticosterone (Sharma et al., 1972). Consistent with the earlier findings these studies also supported the obligatory role of Ca^2+^ in the ACTH-induced steroidogenesis (Perchellet and Sharma, 1979). The conclusions were that cyclic GMP and Ca^2+^ were the two complementary physiological second messengers of the hormone.

The stage was set to directly demonstrate the ACTH-dependent membrane guanylate cyclase in the adrenal cell membranes and also the presence of its companion cyclic GMP system, protein kinase in the bovine adrenal cortex, to strengthen the cyclic GMP second messenger concept.

The cyclic GMP-dependent protein kinase was purified from the bovine adrenal cortex. Its important distinctive characteristic that set it apart from the cyclic AMP signaling system was that its most effective effector molecule was 8-bromo cyclic GMP (Ahrens et al., 1982). Compared to its dibutyryl analog, it was 10,000-fold more active in stimulating the protein kinase activity. This feature established its unique functional identity because the dibutyryl cyclic AMP was more potent than cyclic AMP in stimulating cyclic AMP-dependent protein kinase. Finally, the molecular structure of the cyclic GMP-dependent protein kinase was entirely different from that of the cyclic AMP-dependent protein kinase. The single subunit of the former protein contained both the non-dissociable regulatory and catalytic domains, opposite was the case with the latter protein (Ahrens et al., 1982). Besides providing clarity of the molecular elements of the cyclic GMP signaling system, these unique characteristics of these proteins helped to resolve an earlier argument that cyclic GMP signaling system was the “sub-servant” of the cyclic AMP signaling system *(vide supra)*. Clearly, it was not the case.

Analysis of the adrenal cortex and the adrenocortical carcinoma tissues demonstrated the direct presence of the ACTH/Ca^2+^-dependent membrane guanylate cyclase (Nambi and Sharma, 1981a,b; Nambi et al., 1982). In addition, the distinct features of the membrane and the soluble form of guanylate cyclases were established (Table 1 of Nambi et al., 1982). Most fundamentally, the membrane guanylate cyclase activity was not dependent on the free radical and nitric-oxide-generating agents, including hemin.

These conclusions were supported by the findings of other investigators. ACTH stimulated membrane guanylate cyclase activity in the cultured neurons (Anglard et al., 1985), and ANF peptide hormone stimulated membrane guanylate cyclase activity of several rat tissues (Hamet et al., 1984; Waldman et al., 1984). Ironically, these were the same tissues, in the hands of the same investigators, in which earlier the membrane guanylate cyclase activity was not observed (Goldberg and Haddox, 1977; Murad et al., 1979).

The issue was thus resolved. A hormonally-dependent membrane guanylate cyclase existed in the mammalian cells; it was a transducer of specific hormonal signals and cyclic GMP was their second messenger. In addition, the membrane guanylate cyclase was functionally and kinetically distinct from the soluble form. At this moment, the task at hand was to define the cyclase's molecular nature and to elucidate the mechanism of the hormonal signal transduction.

## Biochemical identity: atrial natriuretic factor receptor, the prototype member of the membrane guanylate cyclase family

A plasma membrane guanylate cyclase was purified from the rat adrenocortical carcinoma and its absolute purity was established by these criteria: (1) protein staining: SDS-PAGE showed a single Coomassie blue- and silver-stained band; (2) isoelectric focusing, native protein and its iodinated form yielded a symmetrical protein peak, which superimposed over the guanylate cyclase activity peak; (3) Western blot analysis using a monospecific polyclonal antibody raised against the native protein yielded a single immunoreactive band which co-migrated with that of the purified protein; (4) the antibody raised against the native protein blocked almost all of its membrane guanylate cyclase activity; and (5) the protein bound ANF peptide hormone in a stoichiometric fashion (Paul, 1986; Paul et al., 1987).

Thus, the protein was pure, it was a membrane guanylate cyclase, and possessed a remarkable characteristic of being also the ANF hormonal receptor. Accordingly, it was named ANF-RGC (ANF RECEPTOR GUANYLATE CYCLASE) also known as GC-A or NPR-A.

The properties of ANF-RGC also resolved the earlier erroneous conclusions on the nature of the membrane guanylate cyclase (Goldberg and Haddox, 1977; Murad et al., 1979). The membrane guanylate cyclase activity was independent of the nitric-oxide-generating agents—catalase, hemin, dithiothreitol, arachidonic acid, and tuftsin, including cigarette smoke. These non-specific activities apparently belonged to the soluble form of the guanylate cyclase whose constituent, critical for the functional operation, was heme (Figure 1).

A parallel report described the purification of a protein from the rat lung and claimed that “ANF-binding and guanylate cyclase activity resided on a single protein” (Kuno et al., 1986). These authors demanded priority on their finding and challenged the priority on the original discovery of ANF-RGC. This issue was settled in a commentary (Sharma, 1988), which revealed that in contrast to the criteria of the absolute purity established for the adrenocortical carcinoma ANF-RGC guanylate cyclase (Paul et al., 1987), the lung guanylate cyclase preparation was crude (Kuno et al., 1986)—in addition to the main and major 120-kD protein it contained at least 13 other minor bands and the authors themselves stated “that it was about 95% pure” (Kuno et al., 1986). The possibility existed that the 5% contaminant contained the separate ligand-binding or the cyclase activity. There were two other serious problems with the claim that the lung guanylate cyclase was also the ANF receptor: (1) it bound only 14.5% of ANF at the noted theoretical value; (2) the lung enzyme was stimulated by hemin, a characteristic of the soluble guanylate cyclase, not possessed by the membrane guanylate cyclase (Kuno et al., 1986). To this date, these deficiencies in the lung enzyme to categorize it as a genuine ANF-RGC have not been resolved.

With two subsequent reports on ANF-RGC purification from the adrenal cortex and its characterization (Takayanagi et al., 1987; Meloche et al., 1988), the concept that this membrane guanylate cyclase is also a surface receptor of the ANF hormone was established. And a new field of the hormone receptor membrane guanylate cyclases was borne.

This happening had a powerful impact on the cellular signal transduction field, as it was a ground-breaker. It demonstrated that the hormonally modulated membrane guanylate cyclase transduction system was radically different from the two existing cyclic AMP and IP_3_ signaling pathways, quoting in the original publication “coexistence of the ANF receptor and guanylate cyclase activities on a single polypeptide chain indicates that the mechanism of transmembrane signal transduction involving mediation by second messenger, cyclic GMP, is different from the well-established adenylate cyclase system. In the hormone-dependent adenylate cyclase, there is an assemblage of individual components—receptor, GTP-binding protein, and catalytic moiety—for signal transduction. In contrast, the presence of dual activities—receptor-binding and enzymic—on a single polypeptide chain indicates that this transmembrane protein contains both the information for signal recognition and its translation into a second messenger” (Paul et al., 1987) (Figure 2).

In the proposed model, ANF-RGC is depicted as a single transmembrane-spanning protein; the ANF-receptor domain resided on the outside, the catalytic domain protruded inside the cell and a transmembrane segment separated its two portions (Sharma et al., 1988; Sharma, 2002; modified version of Figure 1 in Sharma, 2002). Mechanistically, ANF will bind to its receptor which resided in the extracellular domain of ANF-RGC. It will generate a cascade of structural changes, they will be carried over the transmembrane domain of ANF-RGC and finally stimulate its catalytic domain residing in the interior of the cell. The cyclic GMP will be produced and serve as a second messenger of the hormonal ANF signal.

### ANF-RGC family

Almost 3 years after its purification, ANF-RGC mRNA was cloned from the human and rat brain (Chinkers et al., 1989; Lowe et al., 1989), adrenal gland (Duda et al., 1991) and the mouse Leydig tumor cells (Pandey and Singh, 1990). In all cases the deduced protein structure validated the original theoretical prediction (Paul et al., 1987) that ANF-RGC was a single-transmembrane-spanning protein. The transmembrane region divides it in two roughly equal portions: extracellular and intracellular (please note: a more detailed description of a representative membrane guanylate cyclase modular structure is provided in **Figure 4**).

Analysis of the two additional cloned membrane guanylate cyclases demonstrated that they were the surface receptors of the respective hormones, CNP (Chang et al., 1989; Schulz et al., 1989; Duda et al., 1993) and enterotoxin (de Sauvage et al., 1991; Singh et al., 1991); and they have the identical structural topography as ANF-RGC. According to their function, they were named CNP-RGC (CNP receptor guanylate cyclase also known as GC-B or NPR-B) and STa-RGC (enterotoxin receptor guanylate cyclase also known as GC-C). These findings established a three-member surface receptor family of membrane guanylate cyclases, which remains the case to date (reviewed in Sharma, 2010).

### Hormone signaling site resides in the extracellular domain of receptor guanylate cyclases

With the model system of the variant form of ANF-RGC, GCα, cloned from the adrenal cortex, the original study of Duda et al. (1991) demonstrated that the ANF binding site in ANF-RGC resides in the extracellular domain. Compared to ANF-GC, GCα in its structure had only two amino acid substitutions, Gln^338^His and Leu^364^Pro, but it did not bind ANF. Importantly, it did retain basal cyclase catalytic activity. Reconstitution of the native ANF-RGC residues restored the mutant's ANF-binding and ANF-dependent catalytic activities (Duda et al., 1991). Thus, the ANF ligand-binding domain resided in the extracellular domain of ANF-RGC; and one, or both of the residues—Gln^338^, Leu^364^—controlled its ANF-binding and ANF-dependent catalytic activities. Refined point mutation analysis demonstrated that both of these controls resided in Leu^364^ (Duda et al., 1991). Thus, the hypothesis for the ANF signal transduction mechanism evolved. It involved the ANF binding to its extracellular domain in ANF-RGC; the binding initiated a cascade of structural changes in ANF-RGC; these changes, in turn, stimulated its catalytic domain, which resided at the opposite end on the intracellular domain of the cyclase. The generated cyclic GMP served as the second messenger of ANF signal.

This signal transduction mechanistic template was applied to CNP-RGC cloned from the human retina (Duda et al., 1993). CNP-RGC is a homologous protein to ANF-RGC, with 59% structural identity. It was found that Glu^332^ residue of CNP-RGC was critical for the CNP binding and the CNP-dependent catalytic activity of CNP-RGC (Duda et al., 1994). Like ANF-RGC, the site had no influence on the basal guanylate cyclase activity. These studies were extended to compare the identities of the folding patterns of the external domains of these membrane guanylate cyclases (Duda et al., 1994). They were found to be almost identical (Duda et al., 1994). The counterpart of CNP-RGC-Glu^332^ residue is the Gln^338^ residue of ANF-RGC, and that of ANF-RGC-Leu^364^ residue is Val^358^ of CNP-RGC. Conversion of the ANF-RGC residue Gln^338^ to Glu resulted in a change from none to significant CNP signal transduction activity. And, the conversion of Val^358^ to Leu generated significant ANF signal transduction activity in CNP-RGC (Duda et al., 1994). These studies validated that the ligand-binding domain resides in the extracellular domain of ANF-RGC and CNP-RGC, provided identity of the residues that are critical in defining the ligand-binding domains and showed that the folding patterns of these domains in two guanylate cyclases are very similar.

The subsequent crystallization studies on the isolated extracellular domains of ANF-RGC and CNP-RGC validated the conclusions that the hormone-binding site, indeed, resides in the external domain of the guanylate cyclase and Leu^364^ of ANF-RGC and Val^358^ of CNP-RGC constitute critical hydrophobic sites essential for the ANF or CNP signaling but the sites are not the direct hormone-binding sites (Ogawa et al., 2004; He et al., 2001).

### ATP is obligatory for ANF and CNP hormonal signaling

Prior to the molecular characterization of ANF-RGC, two studies with crude membrane preparations proposed that ATP accelerates ANF-dependent ANF-RGC activity (Kurose et al., 1987; Chang et al., 1990). Two different mechanisms for the acceleration were proposed; direct (Kurose et al., 1987) and indirect *via* an accessory protein (Chang et al., 1990). Because the preparations were crude and the guanylate cyclase was uncharacterized, the issue as to which mechanism was correct could not be resolved.

Subsequently, in parallel two independent groups established that ATP is obligatory for the ANF-dependent ANF-RGC transduction activity (Marala et al., 1991; Chinkers et al., 1991). Neither ANF, nor ATP alone, is able to stimulate ANF-RGC catalytic activity. Only when these two are together they are able to stimulate the cyclase. Because the non-hydrolyzable analogs, ATPγS and AMP-PNP, mimicked the ATP effect, both groups proposed that ATP acts directly by allosteric regulation of ANF-RGC (Marala et al., 1991; Chinkers et al., 1991). Later study with CNP-RGC demonstrated that ATP is also obligatory for its CNP-dependent activity (Duda et al., 1993).

The mechanistic models proposed for the ATP effect by these two groups were different, however. Which one is valid, was debated for over two decades. MODEL 1 proposed by the Garbers'group stated “… binding of ANP [ANF] to the extracellular domain of its receptor initiates a conformational change in the protein kinase-like domain (KHD), resulting in derepression of guanylate cyclase activity” (Chinkers and Garbers, 1989). Its central idea was that “KHD in native ANF-RGC suppresses its catalytic module activity; ANF functions by relieving this suppression.” Sharma's group challenged this model on the grounds of their findings that partial or complete deletion of the KHD did not cause any significant elevation in the basal activity of the guanylate cyclase; therefore, ANF signaling did not involve overcoming the KHD suppression of the guanylate cyclase activity, contradicting the central theme of MODEL 1. (Goraczniak et al., 1992; reviewed in Duda et al., 2005b). They proposed an alternate MODEL 2. In it the repressor of the core catalytic domain (CCD) of the guanylate cyclase was the disulfide structural motif of ANF-RGC (Duda and Sharma, 2005). This motif was juxtaposed to the N-terminus of the TM (transmembrane of the domain). The motif repressed the ARM (ATP REGULATED MODULE) domain, which, in turn, inhibited the CCD. ATP-modulated ANF signal overcame the disulfide structural motif-dependent inhibition of ARM resulting in activation of the CCD of ANF-RGC.

The MODEL 2 has been defined in molecular terms by simulating structure of the ARM domain in 3D-terms, experimentally validating the biochemistry of the structure and its physiology through the mouse molecular genetic models (Duda et al., 2001c, 2009, 2013). The definition has been arrived at through biochemical experiments involving point mutation, time-resolved tryptophan fluorescence, Forster Resonance Energy Transfer (FRET), reconstitution, recombinant constructs, molecular modeling, immunohistochemistry and recently mouse molecular genetics (these studies are comprehensively reviewed in Duda et al., 2014).

## Linkage with sensory transduction signals expands the family

Because the property of being a hormonal receptor, transducer and signal amplifier was common in all known members of the membrane guanylate cyclase family (ANF-RGC, CNP-RGC and STa-RGC), the notion was that the cyclic GMP signaling pathway operated solely in the hormonal signaling pathways and was designed to transduce only the signals generated outside the cell. However, it was not the case.

The membrane guanylate cyclase signal transduction paradigm changed with the landmark discovery of the photoreceptor ROS-GC guanylate cyclase. Here the signal initiation occurred inside the cell and the signal transduction system was composed of two separate elements, Signal receiver and the Transducer (detailed reviews Sharma and Duda, 2012; Koch et al., 2010; Pugh et al., 1997).

It was known that in phototransduction, which is the transformation of the light signal into an electrical signal, both [Ca^2+^]_i_ and cyclic GMP are the critical cytosolic regulatory effectors of the photon signal in the vertebrate photoreceptors. Which one of these is the second messenger of the LIGHT signal; how these signals are generated; how they interact with each other; and what was the source of the cyclic GMP was not known, however (early reviews Pugh and Cobbs, 1986; Stryer, 1986; Lamb et al., 1986). Early reports on the successful identification of photoreceptor ROS-GC contradicted each other, and created chaos. They ranged from the guanylate cyclase being a 67 kDa subunit (Horio and Murad, 1991a,b), consisting of “separate regulatory and catalytic subunits” (Stryer, 1991) and of its molecular from of retGC (Shyjan et al., 1992).

The breakthrough came with the seminal observation that a soluble bovine rod outer segment (ROS) fraction stimulated the catalytic activity of a particulate photoreceptor guanylate cyclase in the absence of Ca^2+^ (Koch and Stryer, 1988). This hinted that the guanylate cyclase was of the membrane form; the soluble ROS fraction was Ca^2+^ sensitive and the two components together constituted the phototransduction-linked guanylate cyclase transduction element. However, the conceived structure of this guanylate cyclase was erroneous, believed to be composed of “separate regulatory and catalytic subunits” (Stryer, 1991).

The true identity of the photoreceptor ROS-guanylate cyclase (ROS-GC also known as GC-E) was resolved by establishing its direct purification from the bovine outer segments (OS) (Margulis et al., 1993), the site of phototransduction; and its protein-sequence-based molecular cloning, structure, and function (Goraczniak et al., 1994). ROS-GC had a theoretical molecular mass of 120,360 Da; a value similar to that reported earlier for a bovine (Koch, 1991) and a toad photoreceptor guanylate cyclase (Hayashi and Yamazaki, 1991). Unlike the other known membrane guanylate cyclases, atrial natriuretic factor receptor guanylate cyclase ANF-RGC (Paul et al., 1987; Sharma, 1988; Chinkers et al., 1989; Duda et al., 1991) and type C natriuretic peptide CNP-RGC guanylate cyclase (Chang et al., 1989; Duda et al., 1993), ROS-GC was not hormonally-responsive (Goraczniak et al., 1994). And also ROS-GC was not composed of “separate regulatory and catalytic subunits”; it was not nitric oxide sensitive nor was its structure identical to retGC (Shyjan et al., 1992), which apparently was a cloning artifact whose primary structure was eventually revised to match that of bovine ROS-GC (Accession number M92432).

Hydropathy analysis of its deduced amino acid sequence revealed that topography of this protein was similar to the other three peptide hormone receptor guanylate cyclase members (Goraczniak et al., 1994), i.e., (1) it was a single trans-membrane spanning protein; (2) the TM domain divided the protein into roughly two equal segments, extracellular and intracellular; (3) it showed a significant overall homology with the other peptide hormone receptor guanylate cyclases: 27% identity with STa-RGC, 30% with ANF-RGC, 31% with CNP-RGC. The level of identity rose to 37, 40, and 41% in their intracellular regions, and there were respective identities of 25, 32, and 32% between their kinase like domains. The least identity with peptide hormone receptor guanylate cyclase existed in their extracellular domains: 14% with STa-RGC, 17% with ANF-RGC and 18% with CNP-RGC. Despite these similarities, there were two significant structural variances. The signature ATP-modulated domain Gly-X-X-X-Gly present in ANF-RGC and CNP-RGC was absent in ROS-GC. And the ROS-GC protein beyond the catalytic domain contained a C-terminal extension tail of 90 amino acids, Y^965^-K^1054^, this tail was absent in ANF-RGC and CNP-RGC. On these grounds the discovery of ROS-GC represented expansion and the generation of a new subfamily of the membrane guanylate cyclases.

It is noteworthy that in the membrane guanylate cyclase family ROS-GC is the only one whose molecular identity has been established on the basis of its protein sequence. This strategy has provided an experimental proof for the position of the N-terminus amino acid of the mature protein and demonstrated that the mature protein contains a 56 amino acid N-terminus hydrophobic signal peptide. The theoretical molecular mass of the protein with its signal peptide is 120,361 Da and without it is 114,360 Da. (Goraczniak et al., 1994).

## ROS-GC, a Ca^2+^-modulated two component transduction system

Following the functional description by Koch and Stryer (1988), almost contemporaneously, two independent groups discovered two structurally dissimilar guanylate cyclase activating proteins (GCAPs), GCAP1 (Palczewski et al., 1994; Subbaraya et al., 1994; Gorczyca et al., 1994; Frins et al., 1996) and GCAP2 (Dizhoor et al., 1995). In the presence of free Ca^2+^, did these GCAPs inhibit catalytic activity of the photoreceptor ROS-GC?

Availability of the recombinant (r)ROS-GC made it possible to answer this question. Expressed in the heterologous system of COS cells, ROS-GC was responsive at 10 nM [Ca^2+^]_i_ to the GCAP1 stimulation in a dose dependent manner. The stimulation was inhibited cooperatively by free Ca^2+^ with a K_1/2_ of 100 nM. Under identical conditions, GCAP1 had no effect on the catalytic activity of the recombinant peptide hormone ANF-RGC (Duda et al., 1996b). An important characteristic of the transduction system was that GCAP1 remained bound to ROS-GC at the low and high Ca^2+^ levels, consistent with physiological observations (Koutalos et al., 1995). Similar reconstitution studies established GCAP2 as another Ca^2+^-sensor of ROS-GC signaling. With the subsequent discovery of a second ROS-GC, termed ROS-GC2, or GC-F (Lowe et al., 1995) to distinguish it from the original ROS-GC which was renamed as ROS-GC1 (GC-E), it became clear that a Ca^2+^-modulated system composed of a pair of ROS-GCs and a pair of GCAPs sub-serve phototransduction in the outer segments of photoreceptors. Which of the two ROS-GCs is physiologically linked with phototransduction?

This problem has not yet been solved for ROS-GC2, but by all criteria ROS-GC1 is a vital component of the phototransduction machinery. (1) It is the only one purified directly from the ROS, the site of phototransduction. (2) Direct quantitative estimation of the two isozyme guanylate cyclases in the bovine ROS demonstrates that their ratios are 96% ROS-GC1 and 4% ROS-GC2, suggesting ROS-GC2 presence is minor (Helten et al., 2007). The latest estimation in the mouse shows the same pattern, 76 vs. 24% (Peshenko et al., 2011). Notably, in contrast to the bovine, the mouse study was made with the total OS instead of the isolated membrane fractions. (3) The multiple cone rod dystrophies in humans have only been linked with the ROS-GC1 mutations, none so far with ROS-GC2 (reviewed in Hunt et al., 2010). Finally, (4) it has been demonstrated that the ROS-GC1 gene deletion results sequentially in the cone and then the rod degenerations, indicating that the guanylate cyclase is directly linked with the vitality and the function of photoreceptors (Yang et al., 1999).

## GCAPs interlace Ca^2+^ and ROS-GC

Together with recoverin (Dizhoor et al., 1991; Hurley et al., 1993), GCAPs are the prototype members of the NCS (neuronal Ca^2+^ sensor) proteins family (reviewed in Sharma and Duda, 2012; Koch et al., 2010). Except for S100B, which has two, a general characteristic of this family is that its members have four Ca^2+^-specific structural motifs, termed EF hands. They define the general Ca^2+^ sensor property of the family. While the teleost fish expresses up to 8 forms, the mammals express 2-3 GCAPs (reviewed in: Rätscho et al., 2010).

GCAP1 and GCAP2 are expressed in the same concentration of 3 μM in the bovine ROS (Hwang et al., 2003). Their quantitative, relative, and cellular concentrations in the cones are not known. Yet immunocytochemical studies show that both GCAPs are present in the outer and inner segments of the rods and cones (Gorczyca et al., 1994; Frins et al., 1996). In mouse cones, the expression of GCAP1 exceeds significantly the expression of GCAP2 (Wen et al., 2014). Notably, GCAP1 presence in the cone synaptic pedicles can be clearly defined (Venkataraman et al., 2003).

True to the NCS family trait, GCAPs harbor four EF-hand Ca^2+^-binding motifs, yet only three are functional (reviewed in Koch et al., 2010; Sharma and Duda, 2012). They are second, third and fourth. They sense Ca^2+^ in the range of tens to hundreds nanomoles and are the Ca^2+^-sensor elements of the ROS-GC linked phototransduction machinery.

The knowledge on the [Ca^2+^]_i_ sensing properties of GCAP1 is more advanced than that of GCAP2. In a recent proposal, the transition from the DARK State to the illuminated state of the photoreceptors occurs by the substitution of bound Ca^2+^ with the bound Mg^2+^ to ROS-GC1 (Peshenko and Dizhoor, 2006, 2007). Ca^2+^ associates with GCAP1 with a rate of ~ 2 × 10^8^ M^−1^ s^−1^ (*k_on_*) which is close to the diffusion limit (Sokal et al., 1999). The apparent dissociation constants of each EF-hand for Ca^2+^ are between 0.08 and 0.9 μM and between 0.1 and 1.6 μM in the absence and in the presence of 2 mM Mg^2+^, respectively (Lim et al., 2009). Thus, these nanomolar affinities result in fast dissociation rates (for example, *k_off_* = *k_on_* × *K_D_* = 2 × 10^8^ M^−1^ s^−1^ × 0.2 × 10^−6^ *M* = 40 s^−1^; 1/*k_off_* = 25 ms), consistent with the kinetics of the photoresponse.

GCAP2 senses Ca^2+^ signals differently through its second, third, and fourth EF-hands. They display an apparent *K_D_* of 300 nM (Ames et al., 1999), but no specific assignment for the affinity of each EF-hand has been made so far. It appears, however, that EF hands two and four influence Ca^2+^ sensitivity of GCAP2 more than EF-hand three (Dizhoor and Hurley, 1996). Cysteine accessibility study with GCAP2 mutants shows its restricted reactivity toward Cys^111^ at sub-micromolar Ca^2+^-concentrations, indicating that within the Ca^2+^ concentration range where ROS-GCs are regulated, Ca^2+^-induces conformational changes in GCAP2 (Helten and Koch, 2007). Thus, these fluctuations in the free Ca^2+^ concentrations of the rods and cones control and signal GCAPs to transmit their messages to the bound ROS-GC and be transduced in the generation of cyclic GMP, the second messenger of phototransduction.

### Differential ROS-GC1 modulation by GCAPs

In a significant conceptual advancement, the original study of Krishnan et al. (1998) proposed and demonstrated that “the intracellular region of ROS-GC1 is composed of multiple modules, each designed to mediate a specific Ca^2+^ signaling pathway.” This meant that the sensing mechanisms of [Ca^2+^]_i_ by the ROS-GC1 modules are more than one. This concept was arrived at through the use of ROS-GC1 deletion and ANF-RGC/ROS-GC1 hybrid mutants which retained only the catalytic domain of ROS-GC1. The GCAP1- and GCAP2- modulated domains in ROS-GC1 were separate and they resided on the opposing ends of the catalytic domain of ROS-GC1, GCAP2 at the C-terminal side and the GCAP1 at the N-terminal.

This concept was fine-tuned by mapping the GCAP1- and GCAP2-modulated sites of ROS-GC1 by the use of a comprehensive technology involving soluble constructs of ROS-GC1, direct binding measurements by surface plasmon resonance (SPR) spectroscopy, coimmunoprecipitation and functional reconstitution utilizing progressive deletion constructs and peptide competition studies. The GCAP1 site is composed of two subdomains: transduction, M^445^-L^456^ and binding, L^503^-I^522^ (Lange et al., 1999), and the GCAP2 of one continuous domain, Y^965^-N^981^, containing both the transduction and the binding site (Duda et al., 2005a).

These two GCAP sites have different sensitivities to capture [Ca^2+^]_i_ signals and activate ROS-GC1. The [Ca^2+^] K_1/2_ for GCAP1 is 707 nM and for GCAP2, 100 nM (Hwang et al., 2003). Thus, the latter is about one-order of magnitude more sensitive to [Ca^2+^]_i_ in signaling ROS-GC1 activation.

At a secondary structural level, there also exists a striking difference between the activities of the two GCAPs. The indication is that dimerization of GCAP2 is a necessary requisite to activate the ROS-GC (Olshevskaya et al., 1999). In contrast, the dimeric form of GCAP1 is inactive and does not result in activation of the ROS-GC (Hwang et al., 2004).

## GCAP-modulated ROS-GC1 phototransduction model (Figure 3)

**Figure 3:**
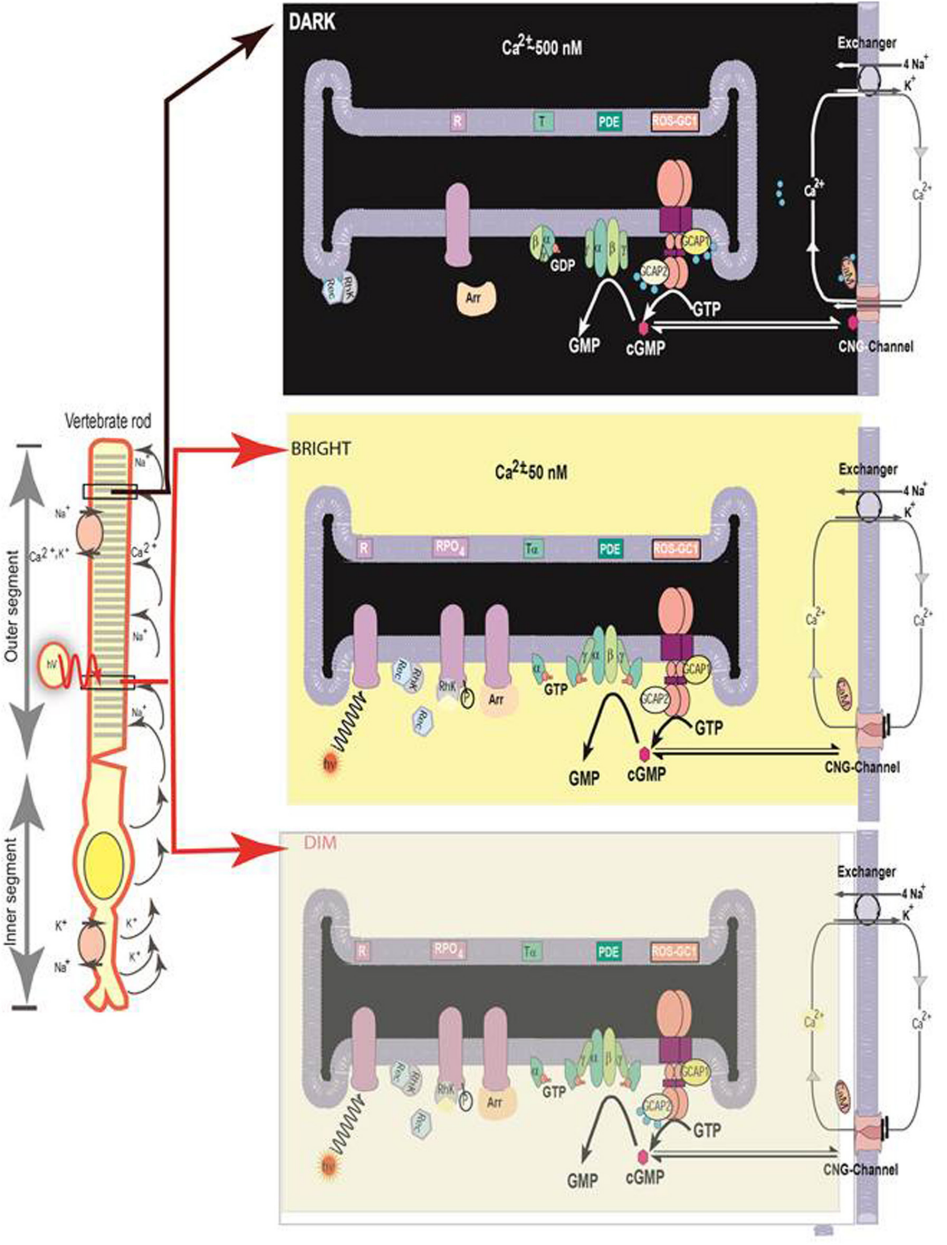
**Schematic representation of the luminosity-dependent operation of the ROS-GC-GCAP transduction system. Left panel**. An illustration of a typical vertebrate rod. In the DARK a circulating current (arrows) is present. It is outward in the inner segment and carried primarily by K^+^; in the outer segment the net charge is inward, with about 90% of the inward flow carried by the Na^+^ and 10% by Ca^2+^ ions. Na^+^/K^+^ exchange pumps in the inner segment membrane and Na^+^/K^+^-Ca^2+^ exchangers in the outer segment membrane (see also right panels) maintain the overall ionic gradients against the dark flows. The capture of a photon (hν) by a rhodopsin molecule in one of the disc membranes of the outer segment initiates the photo-transduction cascade. **Right upper panel. DARK**. The components of the Photo-Transduction cascade are shown in the dark/resting steady-state. Cytoplasmic cyclic GMP, generated by the basal catalytic activity of ROS-GC, keeps a fraction of CNG channels in the plasma membrane open. ROS-GC1 via its ^415^M-L^456^ segment is GCAP1- and via ^965^Y-N^981^ is GCAP2-bound. Ca^2+^ ions enter the cell via the CNG-channel and are extruded via the Na^+^/K^+^, Ca^2+^-exchanger. Synthesis and hydrolysis of cyclic GMP by ROS-GC and PDE, respectively, occur at a low rate. The heterotrimeric G protein transducin is in its GDP-bound state and is inactive. The Ca^2+^ binding proteins calmodulin (CaM), recoverin (Rec) are bound to their target proteins, the CNG-channel, rhodopsin kinase (Rhk), respectively. **Right middle panel**. Absorption of **BRIHT LIGHT** by the visual pigment rhodopsin leads to the activation of the transduction cascade: the GTP-bound α-subunit of transducin activates PDE that rapidly hydrolyzes cGMP. Subsequently the CNG-channels close and the Ca^2+^-concentration falls. The fall in cytoplasmic [Ca^2+^]_i_ is sensed by Ca^2+^-binding proteins: CaM dissociates from the CNG-channel what leads to an increase in cyclic GMP sensitivity of the channel; recoverin stops inhibiting rhodopsin kinase; rhodopsin becomes phosphorylated. Both Ca^2+^-free GCAPs in their changed configurations activate ROS-GC and synthesis of cyclic GMP increases. Arrestin (Arr) binds to phosphorylated rhodopsin and interferes with the binding and further activation of transducin. Enhancement of cyclic GMP synthesis brings it to its original DARK state level and termination of the cascade, which leads to reopening of CNG channels. **Right bottom panel, DIM LIGHT**. The initial fall of [Ca^2+^]_i_ is selectively detected only by GCAP1. In its Ca^2+^-free state GCAP1 attains the activated mode and stimulates ROS-GC activity. GCAP2 remains Ca^2+^-bound and in its inhibitory mode.

The biochemical process by which the rods and cones convert the incoming LIGHT signal into the generation of the electrical signal is termed photo-transduction. Educated by the ROS specific photoreceptor features of the interlocked GCAP/ROS-GC transduction system, a model for its role in operation of the photo-transduction machinery is proposed (Figure 3). This model explains two principal characteristics of photo-transduction machinery, recovery and its variable luminosity dependent operation; the latter characteristic adds an advanced feature to the previous models (Pugh et al., 1999; Burns and Baylor, 2001; Koch et al., 2002, 2010; Sharma, 2002; Luo et al., 2008; Stephen et al., 2008; Wensel, 2008) and depicts the differential involvement of GCAPs in its operation. The luminosity-dependent photo-transduction principles of the present model incorporate a core theme of the previously proposed “Ca^2+^-relay model” (Koch, 2006; reviewed in Koch et al., 2010; Sharma, 2010), which explained the results of a transgenic GCAPs null mice study where the role of GCAP2 was questioned (Howes et al., 2002; Wen et al., 2014).

## Migration patterns and translations of the GCAP signals into production of cyclic GMP are different

After settling the issue that the biochemical modes of two GCAPs in Ca^2+^ signaling of the photoreceptor ROS-GC1 are different and autonomous of each other, the next task was to analyze their migratory pathways at the structural levels, determine the mechanisms involved in transmission and translation of the signals at the CCD of ROS-GC1 into the production of cyclic GMP.

**Figure 4:**
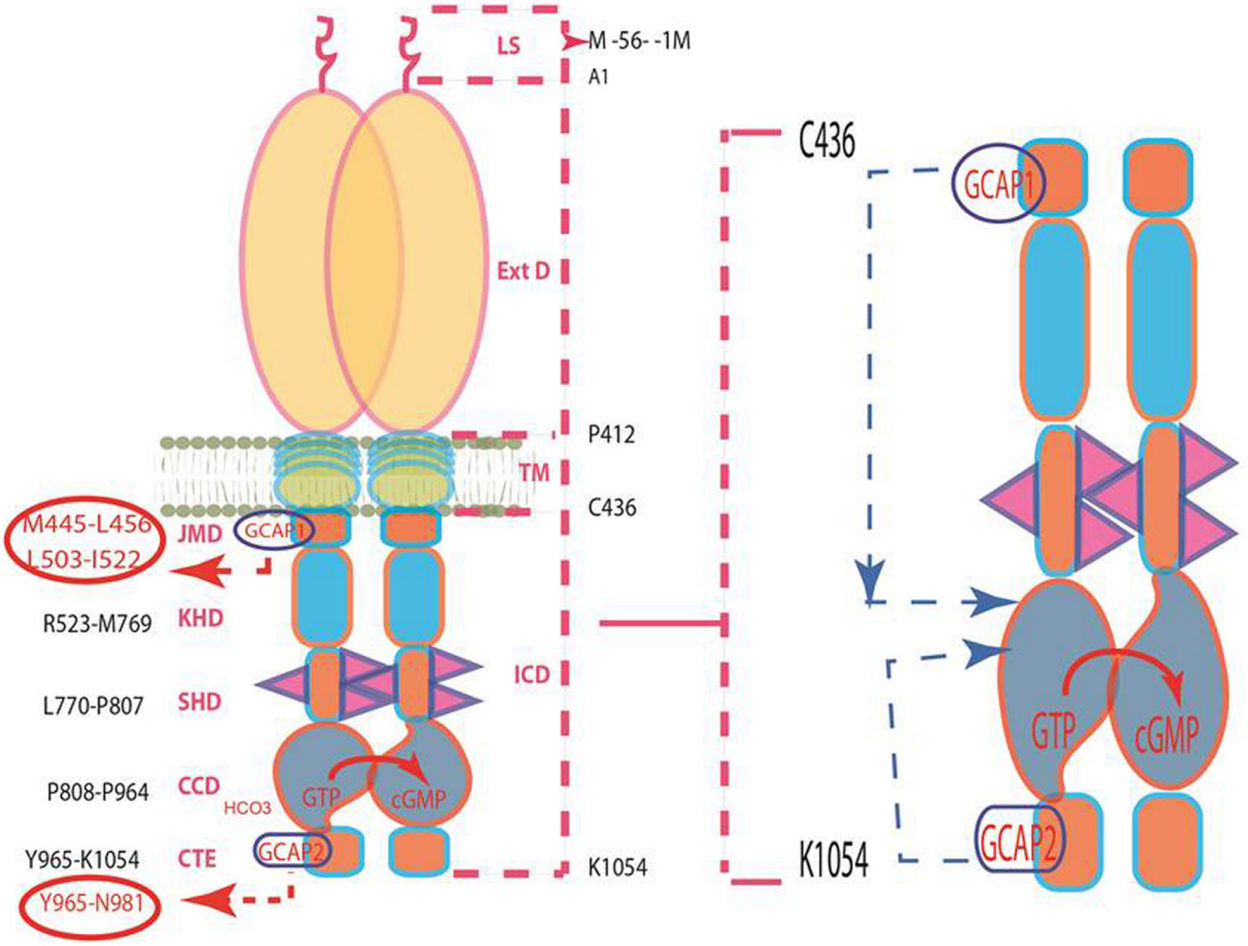
**Pathways of GCAPs signaling of ROS-GC1 activity. Left panel:** Schematic representation of the structural topography of ROS-GC1. It is a single transmembrane homodimer protein. The dashed lines on the right show the defined boundaries of its segments: LS, leader sequence; ExtD, extracellular domain; TM, transmembrane domain; ICD, intracellular domain. All its functional domains are housed in ICD; their designated names and the amino acid residues constituting their boundaries are indicated at the left: JMD, juxtamembrane domain housing the indicated GCAP1-targeted domain; KHD, kinase homology domain; SHD-signaling helix domain; CCD-core catalytic domain; CTE-C-terminal extension housing the GCAP2-targeted domain. It is noteworthy, that the sites targeted by the two GCAPs (encircled) are at the opposite ends of CCD. **Right panel:** The two GCAP pathways run in opposite directions. The trajectory of the GCAP1 pathway shown in blue dashed arrow is downstream. From its origin in the JMD, it passes through the structural domains of KHD and SHD in its course to CCD. In contrast, the trajectory of the GCAP2 pathway (shown in pink dashed arrow) is upstream. From its origin in the CTE, it directly flows to CCD. The CCD exists as an antiparallel homodimer. Both GCAP signals are translated at the CCD into the production of cyclic GMP, which serves the second messenger of the LIGHT signals.

To comprehend these issues and their resolutions, Figure 4 is presented and the parts of the discussion are excerpted (Duda et al., 2012) and briefly recounted. The study involved a programmed domain deletion, expression, *in vivo* fluorescence spectroscopy, and *in vitro* reconstitution experiments. The findings validated (1) in structural terms that operational modes of two GCAPs in signaling of ROS-GC1 activity are different. GCAP1 signal transduction occurs through M^445^-L^456^ and L^503^-I^522^ subdomains of JMD and of GCAP2 signal transduction via the Y^965^-N^981^ subdomain of CTE. These subdomains are at the opposite ends of CCD and they are the sites where the signal of the respective GCAPs originates.

(2) In living cells, both GCAPs by themselves are soluble proteins. Only when they are present with ROS-GC do they become membrane bound through their specific target sites on the ROS-GC1. Thus, ROS-GC1 is the one that bestows on them the property of being membrane bound; the anchoring sites are M^445^-L^456^ and L^503^-I^522^ for GCAP1 and Y^965^-N^981^ for GCAP2. In accordance with these conclusions, disruption of the GCAP1 anchoring site on ROS-GC1 through mutagenesis, results in GCAP1 being a soluble protein. In contrast, GCAP2 remains bound to this ROS-GC1 as its activator. These results prove that the two GCAPs signal ROS-GC1 activation through different modes and the differences reside in the spatial characteristics of ROS-GC1; and because orientations of the two domains of their signal origins are different, their migration pathways are different: GCAP1 downstream from M^445^-L^456^ and L^503^-I^522^ to the P^808^-K^1054^. This assumption was validated through the studies with the SHD-deleted ROS-GC1 mutant. Being downstream from the JMD, the site of the GCAP1 signal, and being a component of the GCAP1 signal trajectory, deletion of the SHD disrupted the GCAP1 signaling of ROS-GC1. But the GCAP2 signaling of the ROS-GC remained intact.

Strikingly, these results also revealed the existence of an in intriguing signaling pathway, never observed before for the membrane guanylate cyclase family. This pathway is unique to GCAP2 and runs opposite to that of GCAP1, upstream from the Y^965^-N^981^ site in CTE to the CCD for translation of the signal into the production of cyclic GMP (Figure 4).

(3) The CCD is a common and conserved translation site for the GCAP1, GCAP2 and all other signals generated by the ligands of the membrane guanylate cyclase family. The current model, based on biochemical analysis, is one in which CCD has intrinsic basal catalytic activity and in its isolated form exists as a homodimer (Venkataraman et al., 2008). Through molecular modeling, this secondary structure has been advanced to its 3D-form, which reveals that its two subunits are in antiparallel conformation (Liu et al., 1997; Venkataraman et al., 2008). Is this model applicable in depicting the 3D structure of CCD in a living cell?

It is. The studies using the bimolecular fluorescence complementation (BiFC) technique demonstrated for the first time that in living cells, the CCD is present in its homodimeric form; and the two subunits are fused in the antiparallel orientations (Duda et al., 2012).

## ROS-GC1 gene linked retinal dystrophies

The disclosure of true identity, complete structure and organization of the ROS-GC1 gene (Duda et al., 1998) and the mechanism by which Ca^2+^ signal modulates its encoded ROS-GC1 enzyme activity made it possible to investigate its role in the human retinal diseases at the genetic levels and explain their biochemistry in molecular terms. For recent comprehensive reviews on this topic the reader is referred to (Hunt et al., 2010; Karan et al., 2010; Sharma, 2010; earlier findings to the year 2002 are covered in Duda and Koch, 2002; Newbold et al., 2002).

In their original study Perrault et al. (1996) demonstrated that there is a point mutation in the human ROS-GC1 gene, F^565^S. Patients inheriting this mutation are inflicted with Leber's congetinal amauresis type 1 (LCA1); they are born blind or become blind soon after birth. Identity of the GCAP1 regulatory regions in bovine ROS-GC1 provided an opportunity to define this dystrophy in molecular terms (Lange et al., 1999). The human F^565^S residue corresponds to the bovine F^514^S residue. This residue is located in the mapped L^503^-I^522^ ROS-GC1 binding region for GCAP1. Biochemical analysis on the heterologusly expressed LCA1-linked ROS-GC1 mutant in COS cells demonstrated that the point mutation causes almost complete loss (84%) of the basal catalytic activity of ROS-GC1 and also of its Ca^2+^ sensitivity to modulation by GCAP1 (Duda et al., 1999b). Thus, the basic structural integrity of ROS-GC1 has been deranged by the mutation and this has also incapacitated GCAP1-modulated Ca^2+^ signaling mechanism of the guanylate cyclase.

Following this lead, similar approach has been used to investigate the second type of retinal disease that correlates with mutations of the ROS-GC1 gene. Named cone-rod dystrophy type 6 (CORD 6), patients suffering with this disease carry one or several point mutations in the dimerization domain of ROS-GC1 (Kelsell et al., 1998). One example is ROS-GC1-E^786^D, R^787^C, T^788^M mutation. The mutation deranges the dimer formation of the guanylate cyclase and reduces the basal catalytic activity of the ROS-GC (Duda et al., 1999a, 2000; Tucker et al., 1999). This basic structural derangement in the guanylate cyclase, intriguingly, increases its sensitivity to GCAP1 and GCAP2. Physiological consequences are that there is a change in the trigger of the Ca^2+^-feedback causing a shift in the response-intensity curve to lower light intensities. These effects explain the photophobia often reported by CORD 6 patients and the accumulating light damage of the retina leading to loss of cone and rod vision over a time period of decades.

## Controlled by low and high levels of [Ca^2+^]_i_, ROS-GC1 is a bimodal transduction switch

Discovered contemporaneously with GCAPs, CD-GCAP (Ca^2+^-dependent guanylate cyclase activator) is the conformational isomer of S100B protein (Pozdnyakov et al., 1995, 1997; Margulis et al., 1996; Duda et al., 1996a, 2002; Wen et al., 2012). In contrast to the commercial S100B that is apparently Zn^2+^-bound, CD-GCAP native to the retinal neurons is Zn^2+^-free (Pozdnyakov et al., 1997).

S100B possesses a striking property toward ROS-GC1 stimulation that is opposite to that of GCAPs. This finding started a new paradigm of Ca^2+^ signaling to the cyclic GMP pathway. Recombinant ROS-GC1 expressed in a heterologous system of COS cells responds at 10 nM [Ca^2+^]_i_ to GCAP1 stimulation in a dose dependent manner. Free Ca^2+^ inhibits this stimulation with a K_1/2_ of 100 nM (Duda et al., 1996a). Zn^2+^-free S100B, however, stimulates native and recombinant ROS-GC1 with a [Ca^2+^] K_1/2_ of 400 nM. Presence and ability of the ROS-GC to couple to GCAPs and/or to S100B places a neuronal cell under stringent [Ca^2+^]_i_ modulation. The cell can accelerate or de-accelerate the generation of cyclic GMP; and in this manner can operate as a Ca^2+^ bimodal switch. In this hypothetical scheme, the bimodal Ca^2+^ ROS-GC transduction switch would be an elegant general mechanism for neural transmissions.

This hypothesis was put to test (Duda et al., 2002). Direct interaction between ROS-GC1 and S100B was demonstrated by cross-linking studies utilizing bis-(sulfosuccinimidyl)suberate. A cross-linked product between ROS-GC1 dimer and S100B occurred. Studies with ROS-GC1 deletion constructs confined an interaction of S100B with the C-terminus of ROS-GC1, aa731–1054. An EC_50_ of 395 nM, calculated using SPR spectroscopy, was comparable to the EC_50_ of 800 nM for stimulation of ROS-GC1 by S100B. Refined analysis revealed that aa952–991 segment of ROS-GC1 defines the S100B binding site with half maximal binding at 198 nM. The site contained a ^966^RIHVNS^972^ motif that was obligatory for binding and a flanking cluster of four aa residues, ^1039^RRQK^1042^ forming a transduction site. This transduction site did not contribute to S100B binding but promoted maximal activation of ROS-GC1.

These interactive physical parameters between S100B and ROS-GC1 were brought to the functional level in the synaptic layers of the retina where the co-presence of ROS-GC1, GCAP1 and S100B existed (Liu et al., 1994; Cooper et al., 1995; Duda et al., 2002). Below 200 nM, Ca^2+^ elicited a dose-dependent decrease in guanylate cyclase activity with an IC_50_ for Ca^2+^ of 100 nM. In contrast, at higher concentrations, ROS-GC activity began to climb with an EC_50_ of 0.8 μM. That GCAP1 is involved in the inhibitory phase of ROS-GC1 activity was confirmed by peptide competition experiments. A sequence motif L^503^-I^522^ in ROS-GC1 is critical and specific for the cyclase activation by GCAP1 (Lange et al., 1999). The core motif within this domain consists of D^507^-R^518^.

Thus, in the synapse ROS-GC1 acts as a bimodal Ca^2+^ transduction switch. GCAP1 has the higher affinity for Ca^2+^ and upon binding, lowers ROS-GC1 activity. S100B captures the high Ca^2+^ signal and upon binding, stimulates ROS-GC1. This ROS-GC1 signal transduction model is schematically diagrammed in (Wen et al., 2012) (Figure 4: Sharma et al., 2014).

### Recent findings

ERG recordings on the retinas of the mouse S100B^−/−^ model suggest that S100B up-regulates ROS-GC1 Ca^2+^-dependent catalytic activity and modulates the transmission of neural signals to cone ON-bipolar cells (Wen et al., 2012).

## Ca^2+^-modulated neurocalcin δ ROS-GC1 transduction system exists in the inner plexiform layer (IPL) of the retina

Besides S100B, a NCS family member, neurocalcin δ (NCδ), is present in bovine IPL (Krishnan et al., 2004; details reviewed in Sharma and Duda, 2012). Co-expressed with ROS-GC1 in the heterologous system of COS cells, NCδ stimulates ROS-GC1 in a Ca^2+^-dependent manner with a K_1/2_ of 0.8 μM. Its target site in the ROS-GC is V^837^-L^858^ (Venkataraman et al., 2008). In accordance with their mutual physiological interaction, NCδ and ROS-GC1 are present together in IPL; added presence of [Ca^2+^]_i_ stimulates native IPL ROS-GC1 activity and nM range of Ca^2+^ keeps NCδ ROS-GC1 bound. The Ca^2+^-dependent NCδ interactive ROS-GC1 kinetic parameters are: without Ca^2+^, NCδ has no affinity for ROS-GC1; in the presence of Ca^2+^, it binds ROS-GC1 with a K_*A*_ of 2.3 × 10^6^ M^−1^ and a *K_D_* of 4.6 × 10^−7^ M.

These analyses reveal that the steps of NCδ binding to and dissociation from ROS-GC1 are Ca^2+^-dependent, they occur within the physiological levels of Ca^2+^, they are direct and of moderate affinity; and they occur within the time span of the visual transduction steps. And significantly, the target site of NCδ for ROS-GC1 is unique to itself; it does not overlap with that of S100B or the GCAPs.

An extraordinary characteristic about the NCδ binding domain in ROS-GC is that it resides directly within the CCD (Venkataraman et al., 2008). This finding demonstrated that NCδ-modulated Ca^2+^ signaling of ROS-GC1 occurs through a new model, its principles are (1) NCδ directly interacts with CCD; (2) it does not require the adjacent N-terminally located α-helical dimerization domain structural element for its interaction; (3) the CCD module, housing the site, is intrinsically active, i.e., it has basal guanylate cyclase activity. (4) The CCD module by itself is dimeric; it does not require the dimerization domain structural element for being so; and (5) the core dimeric form of the catalytic module is directly regulated by the Ca^2+^-bound NCδ; Ca^2+^-unbound NCδ is ineffective (Venkataraman et al., 2008).

## ROS-GC linkage with other than vision-linked neurons

The very brief following sections are meant to demonstrate that the interlocked elements of the Ca^2+^ sensors and ROS-GC family are not unique to just the visual transduction processes they expand their signaling roles to many other sensory and sensory-linked transduction processes.

### Pineal gland

The bovine pineal gland embodies the Ca^2+^-modulated GCAP1- and S100B-modulated ROS-GC1 transduction systems. They exist in two separate sets of pinealocytes. In one set, in the GCAP1 mode, the ROS-GC catalytic activity is modulated by free [Ca^2+^]_i_ K_1/2_ of 100 nM. The second set is in the S100B mode, the ROS-GC activity is modulated by [Ca^2+^]_i_ with K_1/2_ of about 0.5 μM. It has been proposed that these two processes may be involved in the dark and light states of the two types of pinealocytes (Venkataraman et al., 1998, 2000).

### Olfaction

Following the lead observation of Duda et al. (2001b) that GCAP1-modulated Ca^2+^- signaling of ROS-GC1 transduction system is expressed in the rat olfactory bulb, its presence and a role in odorant transduction was investigated (Duda et al., 2001a). Prior to these studies role of the membrane guanylate cyclase transduction system was denied in the odorant transduction and the system was exclusively believed to be “involved in behavioral responses induced by hormones or pheromones, possibly related to reproduction, rather than a response to specific odorants” (Juilfs et al., 1997; Meyer et al., 2000). Yet, these studies revealed the presence of selected components of the membrane guanylate cyclase in the ONE-GC (GC-D) neurons. These neurons were segregated from the dominant odorant linked cyclic AMP signaling neurons.

#### Odorant transduction model (Duda and Sharma, 2009, Figure 4)

A small population of the olfactory receptor neurons (ORN) contains a cyclic GMP signal transduction pathway (Juilfs et al., 1997; Leinders-Zufall et al., 2007; Duda and Sharma, 2008; Pertzev et al., 2010). This pathway resides at the apical region of the cilia. Present in this region is ONE-GC. Its outer domain is a receptor for uroguanylin (Duda and Sharma, 2008). In its inner domain, at the C-terminus, resides the catalytic domain (Duda and Sharma, 2008). The M^880^-L^921^ segment of this domain is bound to Ca^2+^ sensor component NCδ. In the resting state, the ORN is in a 60–100 nM range of [Ca^2+^]_i_ and ONE-GC is in its basal state. The uroguanylin odorant signal starts by its interaction with the receptor domain of ONE-GC (Duda and Sharma, 2008). It is processed through two sequential steps. In step one, ONE-GC is primed and activated minimally. In step 2, [Ca^2+^]_i_ rises. With a K_1/2_ of 0.3–0.8 μM, Ca^2+^ binds NCδ facilitating its interaction with the ONE-GC's segment M^880^-L^921^ (Duda and Sharma, 2008; Duda et al., 2004); Ca^2+^-bound NCδ than signals full activation of ONE-GC and maximal synthesis of cyclic GMP (reviewed in Sharma and Duda, 2010a,b; Zufall and Munger, 2010).

It is envisioned that the operation of step 2 starts with the generation of a small amount of cyclic GMP in step 1. This pool of cyclic GMP opens a limited number of the cyclic GMP-gated channels causing influx of Ca^2+^ in the ORN. Ca^2+^ binds NCδ, which then fully activates ONE-GC.

### Special note

There are clues that, besides NCδ, two additional Ca^2+^ sensors of ONE-GC are involved in the odorant transduction. One is hippocalcin (Hpca) (Krishnan et al., 2009) and the other is GCAP1 (Pertzev et al., 2010). The studies with the Hpca^−/−^ mouse model and with the GCAP1-specific antibodies demonstrate that both Ca^2+^ sensors are physically linked with ONE-GC and are present in the rat and the mouse olfactory neuroepithelium region. In addition, 35% of the total ONE-GC transduction activity is controlled by GCAP1, 27% by NCδ, and 38% by Hpca (reviewed in: Sharma and Duda, 2010a).

### Gustation

Similar to olfaction, gustation (taste) belongs to chemical senses. In response to the sweet, sour, salty, bitter, and umami, taste receptor cells generate electric signal in a process called gustatory transduction (Avenet and Lindemann, 1989; Gilbertson et al., 2000, 2001; Herness, 2000; Lindemann, 1996). The molecular steps in this transduction process are ill defined. Yet in a general scheme Ca^2+^ is pivotal for the process; it depolarizes plasma membrane of the given taste cell, releases the transmitter release and functions through the CNG-gated channels, cyclic AMP and cyclic GMP (Kolesnikov and Margolskee, 1995).

The expression of a Ca^2+^ signaling ROS-GC1 transduction system in the anterior portion of the bovine gustatory epithelium has been demonstrated at the biochemical, molecular and functional levels (Duda and Sharma, 2004). The system is composed of two components: the Ca^2+^-sensor protein, S100B and the transducer, ROS-GC1. Co-immunoprecipitation experiments reveal that ROS-GC1 and S100B physically interact with each other. The precise operational mechanism of this signal transduction mechanism and its physiology has yet not been decoded (reviewed in Sharma and Duda, 2010b).

## Evolution of a general Ca^2+^-interlocked ROS-GC signal transduction concept in sensory and sensory-linked neurons

Extraordinary feature of the ROS-GC signal transduction system is that it is an extremely delicate Ca^2+^-modulated machinery. It is crafted impressively to do so. In a range of nM, [Ca^2+^]_i_ signals produce the ROS-GC activity to its peak level and then they decline it with a [Ca^2+^]_i_ K_1/2_ of 100 nM. In a bimodal fashion, the signals then accelerate it with a Ca^2+^ K_1/2_ of 500 nM to 800 nM. The ROS-GC accomplishes the former mode through its Ca^2+^ sensor partners, GCAPs and the latter function through its CD-GCAP partners. This mechanism of bimodal regulation enables ROS-GC to generate the Ca^2+^-dependent pulsated levels of cyclic GMP, which functions as a second messenger of the sensory and sensory-linked Ca^2+^ transduction mechanisms. Presented illustrations are: (1) LIGHT signal induced GCAP-modulated ROS-GC transduction mode in the photoreceptor cells; (2) bimodal GCAP1 and S100B modes in the photoreceptor-cone synapse; (3) the odorant signal–GCAP1, S100B and Hpca–regulated modes in ONE-GC neurons and (4) not yet well-understood processes of the pineal gland and gustation. Bestowed with this design and clues of its linkage with physiology of the various sensory neurons, these investigators have proposed that “Ca^2+^-modulated ROS-GC” machinery is a central signal transduction component of all the sensory and/or sensory-linked secondary neurons. Accompanied by a nearby CNG channel, the machinery is empowered to de-or hyper-polarize neurons, thereby affecting the generation of action potentials (model in Figure 4 of Sharma, 2010). It becomes an elegant theoretical general mechanism for neural transmissions.

## Conclusion and future directions

This review has briefly chronicled the events that have resulted in the step-by-step development of the field of membrane guanylate cyclase. Its foundation rests on the initial seminal finding made five decades ago (1963) that cyclic GMP exists in rat urine. Based on the conceptual framework of the cyclic AMP second messenger, it implied the existence of a hormonally-dependent membrane guanylate cyclase transduction system that generates cyclic GMP in the mammalian cells. For about a decade, this concept underwent a tortuous evolution; first, the euphoria and then a chaos. Barring ours, and few others, most of the fashionable groups denied existence of this transduction system. The field rekindled with the first purification and characterization of a membrane guanylate cyclase, ANF-RGC. Surprisingly, besides being a guanylate cyclase, it was also a hormone receptor. And, the field of membrane guanylate cyclase was born. Yet, the notion evolved that the membrane guanylate cyclase family is solely represented by its hormone-receptors. The field expanded and changed with the discovery of a Ca^2+^-modulated ROS-GC transduction system. Yet it remained restrictive, believed to be linked with the single physiological function of phototransduction in the photoreceptor cells. This belief also changed with the discovery that besides GCAPs, which are selectively linked with phototransduction, other Ca^2+^ sensors coupled with ROS-GC exist beyond the photoreceptor cells. Consistent with its broader role, Ca^2+^-sensor S100B modulated ROS-GC transduction system is linked with the photoreceptor-bipolar neural transmissions. In addition, the system is also present in the pinealocytes and a ROS-GC-variant (ONE-GC) transduction system is expressed in the rodent olfactory systems.

Exponential expansion of the field has generated new problems which have to be solved. Some of them are briefly outlined below.

The first at hand deals with the modes of guanylate cyclases modulation. Have all the modulators of each cyclase been discovered? Recent studies suggest that it may not be the case as exemplified by ONE-GC. For over a decade this cyclase was considered by some groups an orphan receptor (Fulle et al., 1995; Juilfs et al., 1997) but it is now known that it is the receptor for odorant uroguanylin/guanylin (Leinders-Zufall et al., 2007; Duda and Sharma, 2008; Zufall and Munger, 2010). In this new paradigm, the extracellularly-generated odorant signal needs to be further processed in the intracellular domain of the cyclase in a Ca^2+^-dependent manner (Duda and Sharma, 2009). Thus, ONE-GC becomes the first cross-over functional guanylate cyclase, being modulated by both odorant peptide receptor domain and the Ca^2+^-modulated domain. How many other guanylate cyclases possess this feature and what is the molecular mechanism underlying the process of dual regulation?

Second, a new model of ANF-RGC signal transduction has evolved (Duda et al., 2012). ANF-RGC is the bimodal switch; one switch is the traditional ANF hormone and the other, [Ca^2+^]_i_. [Ca^2+^]_i_ signal targets directly the CCD of ANF-RGC and increases the production of cyclic GMP. What is its mechanism of operation? Are other hormone receptor guanylate cyclases also bimodal switches?

Third, the ANF-RGC receptor subfamily is multifunctional. Besides controlling the well-known processes linked with cardiac vasculature—diuresis, fluid secretion, smooth muscle relaxation, it also influences the processes involved in metabolic syndrome and obesity control (Martel et al., 2010). Similarly, CNP-RGC triggered by its hormonal CNP signal affects such diverse processes as bone growth (Bartels et al., 2004) and axonal sprouting in neurons (Schmidt et al., 2007, 2009). How does it achieve this characteristic?

Transcending to the ROS-GC subfamily, it is also multifunctional; linked with the physiology of visual transduction in the retina, photoentrainment in the pineal gland, as well as odorant- and gustatory transduction. Except for phototransduction, the underlying molecular principles of its linkage with the other sensory processes are lacking.

Fourth, a new model of ROS-GC1 signaling specific to cone photoreceptors has emerged. Its key molecule is the S100B protein. Molecular principles of this model need to be deciphered.

Fifth, ill-defined information suggests that GC-G is linked with the processes of capacitation (Kuhn et al., 2004), atmospheric CO_2_ detection (Chao et al., 2010) and is also modulated by cool ambient temperature (Mamasuew et al., 2008). The molecular explanations for these processes are not available.

Sixth, a common trait of the membrane guanylate cyclase family is that it is a multi-modular protein. Experimentally validated evidence indicates that each of its modular domains is precisely crafted to control the cyclase's functional specificity (Sharma and Duda, 1997). Complete function of each domain is lacking, however. Consequently, none of the intact membrane guanylate cyclase structures have been solved by X-ray crystallography.

Seventh, and final, the basic knowledge gained by the preceding studies will be invaluable in defining the physiology of each of the guanylate cyclases, linking them with respective pathological states, and in the long run, in designing targeted therapies against these pathologies.

### Conflict of interest statement

The authors declare that the research was conducted in the absence of any commercial or financial relationships that could be construed as a potential conflict of interest.
